# Pustular psoriasis triggered by therapy with atezolizumab and bevacizumab^☆^

**DOI:** 10.1016/j.abd.2023.02.003

**Published:** 2023-08-30

**Authors:** Mariani Magnus da Luz Andrade, Guilherme Ladwig Tejada, Juliano Peruzzo, Renan Rangel Bonamigo

**Affiliations:** Hospital de Clínicas de Porto Alegre, Porto Alegre, RS, Brazil

Dear Editor,

Atezolizumab (ATZ) and Bevacizumab (BVZ) are used in the immunotherapy of some advanced tumors.^1^, ^2^ ATZ is an immune checkpoint inhibitor, an antagonist of PDL1, expressed in tumor cells, which allows both the evasion of these cells from the immune system, as well as a reduction in T-cell proliferation.^1^ This class of drugs can cause a variety of cutaneous adverse effects, mainly immune-mediated, such as neutrophilic, bullous dermatoses, and vitiligo.^1^ BVZ is an anti-VEGF, therefore with anti-angiogenic action,^2^ and cutaneous side effects such as exanthema and impaired tissue healing have also been described.^3^

The authors present a rare cutaneous adverse event, pustular psoriasis, after the start of the aforementioned therapeutic combination.

A 55-year-old male patient with a history of mild psoriasis controlled only with topical treatment, presented with erythematous, desquamative plaques with pustules on the dorsum of the hands, elbows, legs, and feet after starting ATZ + BVZ therapy for hepatocellular carcinoma. These medications were administered every 21 days and were infused on the same day. During follow-up, the infusion was withdrawn due to hospitalization because of clinical complications. At the time, there was rapid improvement of the lesions, in four weeks (Fig. 1A). After this period, the infusions were resumed, and psoriasis recurred (Fig. 1B), reinforcing the association between the skin condition and drug administration. The clinical presentation, in the form of erythematous-desquamative plaques with pustules (Figs. 2A and 2 B) and pustule generalization to the trunk (Fig. 3A), associated with the anatomopathological examination (Fig. 3B) with intraepidermal pustules and psoriasiform infiltrate, allowed the diagnosis of psoriatic exacerbation, in the form of pustular psoriasis, triggered by antineoplastic immunotherapy. The case constituted a therapeutic challenge since the patient had an hepatocellular carcinoma developed in cirrhosis due to chronic hepatitis C. Therefore, there was contraindication to the use of drugs such as acitretin and methotrexate, due to the risk of severe liver toxicity (including liver failure), and to the use of anti-TNF, considering the diagnosis of hepatitis C. Moreover, the patient developed arthritis in the interphalangeal joints, reinforcing the choice of systemic therapy. Considering these limitations, the authors chose an interleukin (IL-) inhibitor, ustekinumab (anti-IL 12/23).

**Figure 1:**
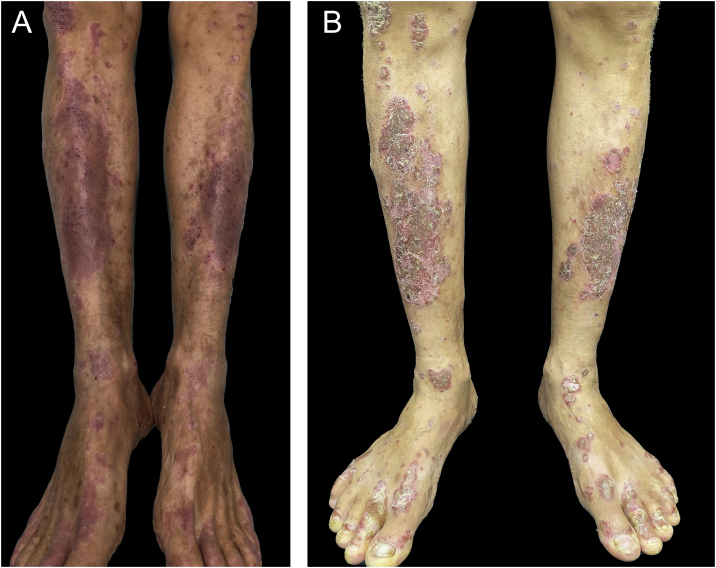
(A) Patient showing improvement of psoriasis lesions after withdrawal of atezolizumab and bevacizumab infusion; (B) Patient showed recurrence of psoriatic lesions after a new infusion, as erythematous-desquamative plaques with pustules.

**Figure 2:**
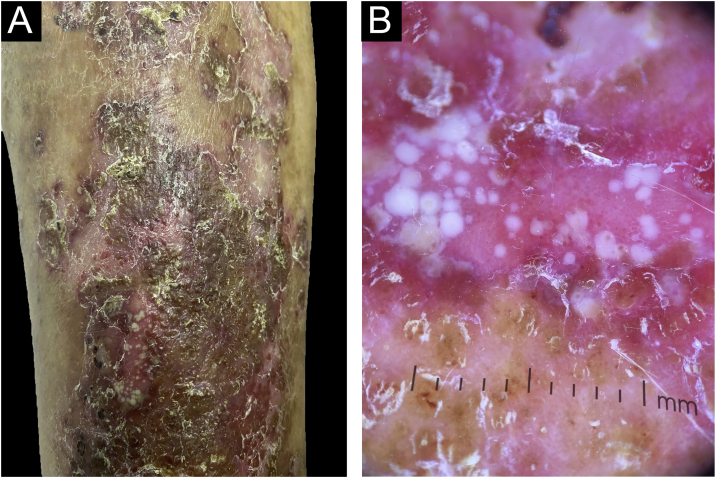
(A) Detail of the clinical lesions on the legs, showing erythematous-desquamative plaques with pustules; (B) Dermoscopy showing the pustules.

**Figure 3:**
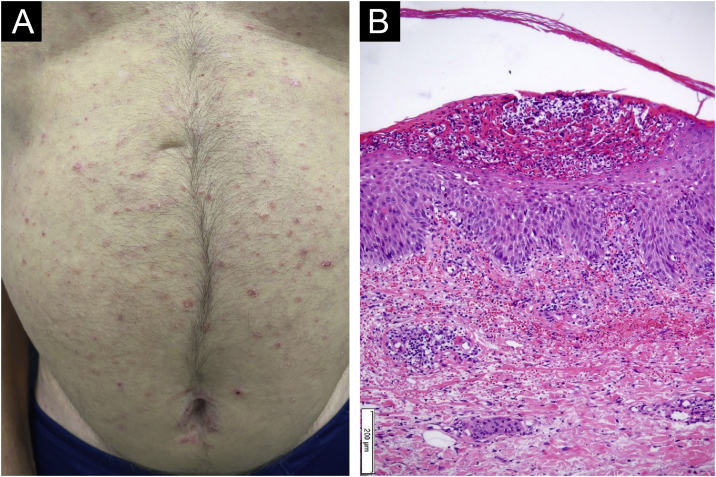
(A) Presence of generalized pustular lesions on the trunk; (B) Anatomopathological examination showed intraepidermal subcorneal pustules and psoriasiform infiltrate (Hematoxylin & eosin, ×100).

Interestingly, there have been reports of psoriasis improvement with the use of BVZ.^4^, ^5^ Vascular proliferation in the papillary dermis is known to play an important role in the pathophysiology of psoriasis.^2^ Moreover, it was found that VEGF levels are higher in psoriatic lesions when compared to healthy skin. Plasma factor levels have also been observed to be higher in patients with psoriasis than in healthy ones.^2^

On the other hand, ATZ seems to induce a pro-inflammatory state, with a change in the cytokine profile, with an increase in TNF-α and IL-17 levels, which would explain psoriasis onset or worsening.^1^ Skin changes due to ATZ usually occur within five to nine weeks after beginning therapy.^1^ In the present case, psoriasis worsening occurred nine weeks after starting therapy, in agreement with the literature. Guttate, inverse, and palmoplantar presentations of psoriasis exacerbations have been described with the use of ATZ.^1^ Therefore, the case highlights the combination related to the eruption, and also the unusual pustular presentation.

## Financial support

None declared.

## Authors' contributions

Mariani Magnus Andrade: Collection, analysis and interpretation of data; drafting and editing of the manuscript or critical review of important intellectual content; critical review of the literature.

Guilherme Ladwig Tejada: Collection, analysis and interpretation of data; drafting and editing of the manuscript or critical review of important intellectual content; critical review of the literature.

Juliano Peruzzo: Analysis and interpretation of data; critical review of important intellectual content; intellectual participation in the propaedeutic and/or therapeutic conduct of the studied cases; critical review of the literature.

Renan Rangel Bonamigo: Analysis and interpretation of data; critical review of important intellectual content; intellectual participation in the propaedeutic and/or therapeutic conduct of the studied cases; critical review of the literature.

## Conflicts of interest

None declared.
